# Type I IFN deficiency: an immunological characteristic of severe COVID-19 patients

**DOI:** 10.1038/s41392-020-00306-4

**Published:** 2020-09-14

**Authors:** Zhenling Wang, Hailong Pan, Boguang Jiang

**Affiliations:** 1 State Key Laboratory of Biotherapy and Cancer Center, West China Hospital, Sichuan University and Collaborative Innovation Center of Biotherapy, Chengdu, 610041 China; 2 Department of Quality Management, China National Biotec Group Company Limited, 100020 Beijing, China

**Keywords:** Infectious diseases, Infection

**Fig. 1:**
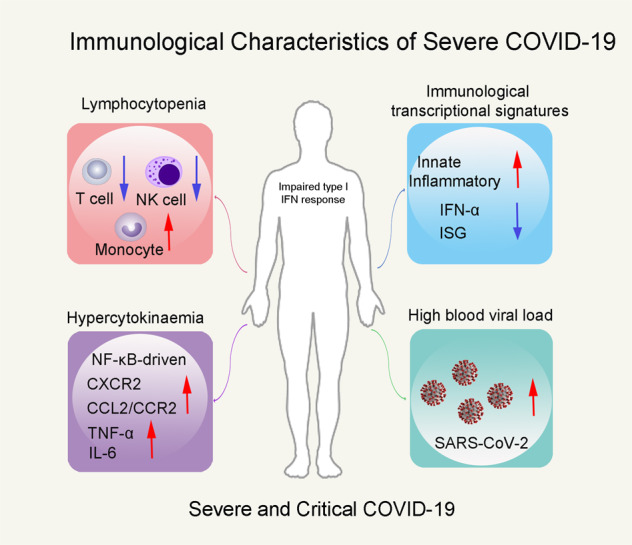
Immunological characteristics of severe COVID-19 patients. Impaired type I IFN response featured immunological characteristics of severe COVID-19 patients, accompanied by lymphocytopenia, hypercytokinaemia, and high blood viral load.

Recently, a paper published in *Science* by Hadjadj et al. reported that type I interferon (IFN) deficiency, could be a hallmark of severe coronavirus disease 2019 (COVID-19) caused by severe acute respiratory syndrome coronavirus 2 (SARS-CoV-2). Severe COVID-19 was also associated with a lymphocytopenia, persistent blood viral load, and an exacerbated inflammatory response (Fig. 1). These findings provide insights into the treatment of severe COVID-19 patients with type I IFN.^1^

Severe COVID-19 is clinically characterized by two-phase disease progression.^2^ A secondary respiratory worsening 9–12 days after the first onset of symptoms can occur early in the disease. Among the respiratory failure patients, young adults (aged 50 years and lower) with previously mild comorbidities have a relatively high rates.^3^ The respiratory failure is concomitant with characteristic CT scan, lymphocytopenia, high prothrombin time, and D-dimer levels.^4^ This biphasic evolution suggests a dysregulated inflammatory host response driven by virus resulting in an imbalance between pro- and anti-inflammatory mediators.^5^ However, the immunological features and mechanisms involved in COVID-19 severity are unclear. In order to test whether the severity disease can be caused by SARS-CoV-2 viral infection and hyperinflammation, Hadjadj et al. conducted a comprehensive immune analysis of grouped 50 COVID-19 patients with different disease severity.

First, to identify whether the severe disease induced lymphocytopenia, Hadjadj et al. compared the peripheral blood leukocytes density of variously severe patients by combining mass cytometry with visualization of high-dimensional single-cell data based on t-distributed stochastic neighbor embedding. There is a significantly decreased density of NK cells and CD3^+^ T cells in severe and critical patients, while the density of B cells and monocytes was increased. The authors determined the functional status of specific T-cell subsets (CD4^+^/CD8^+^) and NK cells based on the expression of activation (CD38, HLA-DR) and exhaustion (PD-1, Tim-3) markers. They observed that the activated NK and CD4^+^/CD8^+^ T cells were increased in all infected patients, while the exhausted CD4^+^/CD8^+^ T cells and NK cells were increased in only severity patients. This result supported lymphocytopenia correlates with disease severity.

Subsequently, Hadjadj et al. characterized the disease severity by studying immunological transcriptional signatures, the expression of 574 immune-related genes in peripheral white blood cells was quantified by direct probe hybridization. And the differentially expressed genes evaluating severity grades were identified. Patients with various disease severity were separated by principal component analysis and the severity-related genes were enriched by GSEA (*q* value < 0.2). A severity-dependent manner was found in activation level of the innate and inflammatory pathways. IFN responses were crucial in anti-viral immune, but were tested relatively lower in severe cases of COVID-19. Here, in critical COVID-19 patients, the genes involved in type I IFN signaling (such as IFNAR1, JAK1, TYK2) were upregulated, whereas the IFN-stimulated genes (ISGs) (such as MX1, IFITM1, IFIT2) were dramatically downregulated. Compared with patients that had mild-to-moderate infection, the ISG score (based on the mean expression value of six ISGs defining a type I IFN signature) was significantly reduced in critical patients. Being consistent with ISG scores, IFN-α2 protein in plasma was significantly lower in critical than in mild-to-moderate patients by Simoa digital ELISA. ISG score and plasma IFN-α2 from blood collected prior to respiratory failure revealed that the low type I IFN response preceded clinical deterioration to critical status. Then, the global type I IFN activity was measured by an in vitro cytopathic assay (protection of Madin–Darby bovine kidney cells against cell death after infection with vesicular stomatitis virus). There is a lower IFN activity in serum of severe or critical patients than that of mild-to-moderate patients. They next stimulated whole blood cells with IFN-α and observed a comparable increase in ISG score in any groups. Among these patients, there is a higher blood viral load in severe and critical patients evaluated by digital PCR. By correlated analysis of viral loading with IFN-α production either on protein or on gene level, the authors suggest that the most severe cases of COVID-19 are featured by impaired IFN-α production.

Hypercytokinaemia observed in severe COVID-19 cases is characterized by increased plasma levels of pro-inflammatory cytokines.^5^ Thus, interleukin (IL)-6, tumor necrosis factor (TNF)-α, IL-1β, and IL-10 proteins in the plasma of patients were quantified using Simoa ELISA assay. IL-6 and TNF-α were detected with high levels in severe and critical patients compared to mild-to-moderate patients. While IL-1β and IL-1α were not detected. This study suggests that severity of COVID-19 was characterized by increased TNF-α and IL-6 production.

To further explore the transcription factors that may cause the excessive inflammatory response of COVID-19, the authors observed that upregulated genes in severe or critical patients mainly belonged to the NF-κB pathway by a kinetic analysis. Aberrant NF-κB pathway activation resulted excessive innate immune sensor activation by pathogen-associated molecular patterns (PAMPs) and damage-associated molecular patterns (DAPMS). Two markers of cellular necrosis and damage, namely LDH and receptor-interacting protein kinase-3, were found to be involved in disease severity and significantly elevated in patients with severe COVID-19. While mounting specific immune response, target organ infiltrations of neutrophils and monocytes could be facilitated by overproduction of chemokines thus contribute to development of acute respiratory distress syndrome. Therefore, the expression of chemokines (CXCR2, CXCL2) and chemokine receptors (CCR2, CCL2) of neutrophils and monocytes was detected. Neutrophil chemokine receptor CXCR2, monocyte chemotactic factor CCL2, and receptor CCR2 were significantly upregulated in severe and critical patients. Overall, these results provide a framework of ongoing inflammatory cascade that impaired type I INF production and high viral load perhaps fueled by both PAMPS and DAMPS.

Overall, Hadjadj et al. identified an impaired type I IFN response, characterized by no IFN-β and low IFN-α production and activity, should be a hallmark of severe COVID-19. Clinically, type I IFN deficiency is associated with hyperinflammation driven by NF-κB and lower viral clearance. The authors indicated that IFN response is possible to incorporate as an indication to assess early severe COVID-19. The application of IFN administration and targeted anti-inflammatory therapies may aid in the development of improved treatments to overcome SARS-CoV-2 infection.
